# Textile Supplies for Hospitals

**Published:** 1916-01-22

**Authors:** 


### TEXTILE SUPPLIES FOR HOSPITALS

### III. The Raw Materials: Cotton and Linen.

All considerations of the quality of goods lead back to the raw material from which they have been made, and fine distinctions of quality involve differences of material difficult to trace. The broader divisions present no insuperable difficulty, while the broadest are recognisable with ease. The great natural division between fibres is in accordance with their origin—animal or vegetable. One mineral fibre exists and is spun into coarse yarns and even woven into rude fabrics, but this material, asbestos, may be regarded as belonging to a separate sphere. The materials are animal, like wool, hair, and silk; or vegetable, like cotton, linen, hemp, jute, and the more rarely met ramie. The so-called artificial silks are vegetable in origin also, prepared by the liquefaction of cellulose derived whether from cotton or wood-pulp, and if these are not in precise terms raw material, and not encountered frequently in hospital practice, their existence may at least be mentioned. Apart from the numerous large and small differences distinguishing animal from vegetable fibres is one of practical moment useful in an emergency. In discriminating between one origin and the other, nothing is more usual than to test samples with a flame and to note their behaviour in burning. The vegetable fibres as a whole are readily inflammable, and ramie is most noticeably so. A bead of fire runs quickly along a detached thread of cotton yarn, little odour is given off in burning, and the ash is unmistakably a fairly pure carbon. The animal fibres do not so burn. A thread or fibre of wool shows little inclination to convey flame; it gives off an ammoniacal smell and swells as it chars. Silk is even less apt to burn than wool, and diffuses an unpleasantly fatty odour when held in the flame. Thus in a rough way the animal fibres may be distinguished as those which do not burn.

### The Characteristics of Cotton.

Cotton in one form or another is in some senses the most important of all fibres. It is the most largely grown and the most cheaply manufactured, with a legion of recognised uses and with capabilities that are being expanded from year to year. It arises as a seed-hair attached to the outside of the globular cotton-seed, and has as its natural function the dissemination of the cotton plant. Several seeds are enclosed in a capsule or ball which at the time of ripening opens to expose the fibre within. The produce is plucked and conveyed to the ginnery, where the cotton is stripped from the seed by the revolution of rollers usually furnished with saw teeth which are too often responsible for damage to the delicate seed-hairs. The seed, when pressed at the oil-mills, yields a substitute for olive-oil and a residual oil-cake for cattle, while the cotton, after being baled by the ginner, is re-compressed into tighter bales subsequently for shipment. The cotton plant secretes oil in its seed, and a proportion of oily or waxy matter is laid as a preservative upon the cotton fibre. This natural wax that is arduously removed by the bleacher is water-repellent in its nature, and cotton-fibre deprived artificially of its wax becomes the absorbent cotton of the surgeon. With the waxes are associated protoplasmic matter or pectose and the dingy colouring matter of unbleached cottons. Together these superficial deposits amount normally to something less than 1 per cent. of the weight of American cotton, and with a further trace of mineral matter they represent the chemical constitution of cotton, which is almost pure cellulose, holding under natural conditions some 8 per cent. of water. The purity of the cellulose of cotton makes the material desirable for other purposes than the dressing of wounds, and notably for gun-cotton and the propulsive explosives at large. In time of war the alternative demand amounts to an inconvenience for the explosives manufacturers, and the surgical-dressing manufacturers are in competition for the same article, which is not the cotton removed from the seed by the cotton gin, but that which remains adherent to the seed after ginning. This residue is less desirable by the spinner than the commercial crop, and is sold at a cheaper rate under the name of "linters."

Cotton, or, at all events, cultivated cotton, is valuable to the spinner and manufacturer of fabrics for a reason that has little to do with its chemical constitution. This uni-cellular seed-hair, upon arrival at maturity, and on storage, dries, and does not do so evenly, with the result that the individual filament flattens and acquires a natural twist. It is due to this feature that isolated fibres of cotton can be bound firmly into a continuous and fine thread. The twist of the filaments lends them their cohesive power, and absence of this small but immensely important feature disqualifies some seed-hairs which might otherwise seem capable of filling the same place as cotton. The bulk in which it is obtainable, the uniformity of the growth and the economy of all pertaining to its handling and manufacturing, when taken in conjunction with its natural properties, make cottton the intimately familiar fibre that it is in all directions. Raw cotton is of kinds more numerous than need be detailed, ranging from the Sea Island cotton, from which gossamer yarn can be spun, far exceeding in commercial value the costliest silk, down to materials almost too poor to be reckoned as cotton, which find their sole employment in combination with rag wool. The two descriptions of practical importance are the Egyptian and the American growths. The former, in the raw state, are mainly brown in tint, of a longer staple than ordinary American, and are used for fine spinning and for the goods generically called, after the town

Note.—Previous articles in this series appeared on Dec. 18, 1915, and Jan. 8, 1916. The next article will treat of "Wool and the Animal Fibres."

of their origin, Bolton cloths. For sheets, towels, aprons, and print uniforms the hospitals rub acquaintance almost solely with American cotton, and in the cheapest sheets and heavy cotton coverlets, with at least a portion of American virgin cotton in its second-hand condition as cotton-waste.

### Linen Fibre.

Until the seventeenth century cotton occupied an altogether obscure place in Western textile economy, and at materially more recent dates it appeared as the usurper in place of the indigenous flax. Considering the similarity of finished cotton and linen fabrics, the contrast between the seed-hair of some such length as one inch, and flax the least fibre of any length up to thirty inches, is striking enough to compel notice. The one is recovered easily from the berries, where the other is got, with much more trouble, from between the stem and the bark of *Linum usitatissimum*. Deprived of the wax and resin that form something like one-fourth of the weight of unbleached flax, linen is a cellulose as pure as that of cotton. Its fibres are multi-cellular and bound together with pectose, and they are capable of greater or less division into ultimate filaments. The same plant provides the fibre as furnishes linseed; but flax grown for fibre is torn from the ground before the seeding is complete. The stems are assorted and bound into sheaves, and left to dry. After a delay extending with advantage up to twelve months, the stalks are "retted," or subjected to a process of fermentation designed to soften the agglutinants and loosen the fibre from the straw. Differences in the colour of raw flax arise from the methods pursued in retting, a grey shade resulting from retting in dew or stagnant water and a golden colour from treatment in running water. The process having gone far enough, the sheaves are air-dried and free from their woody concomitants by treatment in scutch-mills.

Between common and the finest flax there are differences of commercial value ranging up to 500 or 600 per cent., and in comparison with cotton the fibre is in general respects irregular. The longest, finest, and best coloured fetches most money, and the middle part of each stem, shorn of the tip and the root ends, is taken to make the best goods. "Long line," a term descriptive not so much of the flax as the method of its preparation in manufacture, signifies manufacture from flax-middles uncut. "Cut line" is flax middles cut into two for convenience of manufacture. "Tow" is the short and imperfect fibre removed from the longer in the course of dressing, and tow is a name used impartially to distinguish the short filaments of any of the best fibres—flax, hemp, or jute. Tow from flax and jute is a less important surgical dressing in these days than the omnipresent cotton-wool, and tow is used also in manufacture. An inferior linen for towels and bed-ticks is spun and woven from tow, and is characterised by greater lumpiness than qualities made from long flax. An advantage held by linen over cotton lies in the strength derived from the greater length of its fibres, and this is one that has to be paid for in the higher rate of price. Linen yarns may be spun to great fineness, but they do not attain the uniformity of cotton, and in looking at materials in the finished, and not the raw, state this want of evenness serves as a tell-tale. Thus in a union fabric with linen warp and cotton weft it will be found commonly that the variations of thickness in the yarn, seen in holding the sample before the light, clearly denote the one from the other. The test is not infallible, and there are circumstances in which linen and cotton are hard to distinguish one from the other with final certainty, but it can be taken generally that in all-linen cloth "slubs," or thick places in the thread, will be found running in both directions of the cloth. It is largely in goods capable of being made either with linen or cotton that flax is used in the hospital service, and the distinctions warrant closer attention later.

### Ramie and Flax.

The virtue of the finest linens lies in their beauty in addition to their strength, and the coarse flax used for rough and heavy fabrics like canvas has rivals in coarser bast fibres than itself. There was a time when hemp was made into shirts and sheets, and even into breeches, although outside rope and canvas it has few uses to-day. Hemp is in effect a coarser flax and one that has been overshadowed in practical importance by the cheaper rival, jute. In a carpet from which long wear is expected a manufacturer will employ as his binding thread strong flax, and for the backing, which is not subjected to wear, takes jute, as does the linoleum manufacturer. In jute the cellulose is of a more ligneous nature than in the superior bast fibres, and although there is jute supple and glossy enough to be used in decorative fabrics its main function is to provide wrappers.

Ramie fibre, derived from the side of the bark of a plant of the nettle species grown principally in the East, belongs to the order of things more often heard of than seen if its uses in the manufacture of incandescent gas-mantles and undergarments of reputed hygienic properties be excepted. The commercial manufacture of the article has produced many failures and strikingly few successes, and, presumably, these may be traced to certain congenital defects. The strength of a ramie thread measured by a direct pull is extraordinary, and in excess probably of any thread of equal length per pound. The feature would seem to single out this material for the hard usage of public institutions, but the advantage is an illusory one. Ramie is almost devoid of elasticity, its threads break with remarkable ease at any knot made in them, and its fabrics crack when they are folded and rolled in the laundry. The high inflammability of the material, making it dangerous to use in proximity to lights, has been mentioned in brief, but no account of its peculiarities would be complete without noting that, in contradistinction to the fibres in general, ramie does not contract when wet. A closely woven cotton will become water and air proof when thoroughly wetted, whereas a cloth manufactured from ramie yarn remains open at its pores.

